# The Power of Boolean Implication Networks

**DOI:** 10.3389/fphys.2012.00276

**Published:** 2012-07-23

**Authors:** Debashis Sahoo

**Affiliations:** ^1^Institute of Stem Cell Biology and Regenerative Medicine, Stanford UniversityStanford, CA, USA

**Keywords:** bioinformatics, cancer, computational biology, differentiation, microarray analysis, prognostic biomarkers, stem cell, systems biology

## Abstract

Human diseases have been investigated in the context of single genes as well as complex networks of genes. Though single gene approaches have been extremely successful in the past, most human diseases are complex and better characterized by multiple interacting genes commonly known as networks or pathways. With the advent of high-throughput technologies, a recent trend has been to apply network-based analysis to the huge amount of biological data. Analysis on Boolean implication network is one such technique that distinguishes itself based on its simplicity and robustness. Unlike traditional analyses, Boolean implication networks have the power to break into the mechanistic insights of human diseases. A Boolean implication network is a collection of simple Boolean relationships such as “if A is high then B is low.” So far, Boolean implication networks have been employed not only to discover novel markers of differentiation in both normal and cancer tissues, but also to develop robust treatment decisions for cancer patients. Therefore, analyses based on Boolean implication networks have potential to accelerate discoveries in human diseases, suggest therapeutics, and provide robust risk-adapted clinical strategies.

## Introduction

In the past detailed single gene investigations in the context of human diseases was extremely successful and produced many useful drugs (Miller et al., 1982; Slamon et al., 2001; Cunningham et al., 2004; Scott et al., 2012). However, the progress was extremely slow and the success was achieved at the cost of a huge number of failed investigations with multiple billions of dollars in investments (Arrowsmith, 2011; Allison, 2012). Unlike in the past years, it is now easy to gather information from tens of thousands of genes simultaneously. Modern approaches can leverage these huge amounts of biological data to understand human diseases. Therefore, a recent trend in analysis has been shifted to multiple genes that are part of a single functional unit commonly known as networks or pathways. The new approaches have been termed network analysis or systems biology. Clearly, these new approaches have the potential to tackle the complexity of human diseases (Mootha et al., 2003; Segal et al., 2003; Basso et al., 2005; Subramanian et al., 2005; Margolin et al., 2006; Bonneau et al., 2007; Lee et al., 2009; Schadt et al., 2010; Bousquet et al., 2011; Gupta et al., 2011; Jornsten et al., 2011). However, the systematic noise in the system has always challenged these approaches. The noise in the system is due to experimental or biological noise and also noise in measuring gene expression values in a microarray hybridization experiment. In addition to noise, other challenge to the network-based approaches is to translate the discoveries to the clinic.

**Figure 1:**
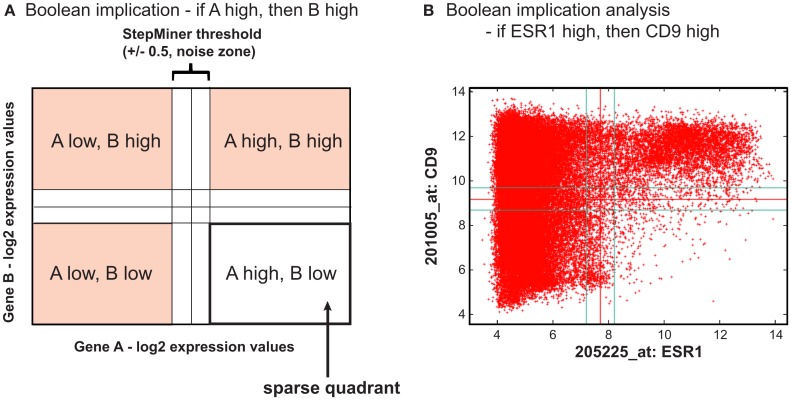
**Boolean implication in gene expression database**. Boolean implication is a pair-wise gene expression relationship between two genes with respect to their gene expression values. **(A)** Schematic example of a Boolean implication between two genes A and B. Threshold to separate high and low gene expression values are computed using StepMiner. A noise margin of 0.5 is used for statistical calculations. Each of the four quadrant is tested for their sparsity. In this case, A high and B low quadrant is sparse representing the Boolean implication “if A high, then B high.” **(B)** An example of a significant Boolean implication between ESR1 and CD9: if ESR1 high, then CD9 high. Every point is a microarray experiment performed on human samples on Affymetrix platform. There are 46,045 microarrays in this scatter plot all of which were downloaded from NCBI’s Gene Expression Omnibus (GEO) website.

In this mini review, we discuss a systems biology or network-based analysis using Boolean implication network (Sahoo et al., 2008). A Boolean implication network is simply a collection of Boolean implication relationships as described by Sahoo et al. (2008). Boolean typically means a logic calculus of two values, which are high and low gene expression values in this context. A Boolean implication relationship is a simple “if-then” relationship between the high and low gene expression values between a pair of genes. For example, “if A is high, then B is high” is a Boolean implication relationship between a pair of genes A and B, where A high and B low is ruled out as a possible scenario as shown in Figure 1. Therefore, whenever gene expression of A is high, we observe gene expression of B is also high. In other words, A high is a subset of B high. In a two dimensional scatter plot between two genes and their thresholds for high and low values, there are four possible quadrants: “A low B low,” “A low B high,” “A high B low,” and “A high B high.” One or more sparse quadrants in this plot is mathematically represented as a Boolean implication. For example, the Boolean implication “if A high, then B high” represent a sparse “A high B low” quadrant. There are six possible Boolean implication relationships, two of them are symmetric, and other four are asymmetric. The symmetric Boolean implication relationship has two diagonally opposite sparse quadrant and the asymmetric Boolean implication relationship has only one sparse quadrant. As shown in Figure 1, the threshold to define “high” and “low” gene expression levels are determined using StepMiner (Sahoo et al., 2007). The expression levels of each probeset are sorted and a step function fitted (using StepMiner) to the sorted expression level that minimizes the square error between the original and the fitted values. We determined the noise margin by using very tightly correlated genes and found that there is still a difference of twofold change (in log scale a value of Miller et al., 1982) among the values that are linearly related. Therefore, we used a noise margin of 1 (threshold −0.5 to threshold +0.5) and discarded all the microarrays that fall within these region for Boolean implication analysis. The noise margin was an important consideration that allowed us to identify many significant Boolean implication relationships.

## Systems Biology Using Boolean Implication

It is possible to discover Boolean implication relationships in the largest possible dataset that include all publicly available microarrays from Gene Expression Omnibus (GEO) or ArrayExpress. These relationships represent natural invariants in a particular species. For example, a Boolean implication relationship in a particular dataset that contains all human samples on Affymetrix platform represents a natural invariant gene expression relationship in human. Many of these invariants are due to tissue specific gene expression. For example, a brain specific gene and a prostate specific gene can never be expressed together. Therefore, they will have a Boolean relationship of the form “if A high, then B low.” Similarly, many of these relationships can be due to developmental gene expression pattern or related to the biological process of differentiation. Mining developmentally regulated genes (MiDReG) is a simple algorithm that uses Boolean implication to identify genes expressed at different stages of differentiation (Sahoo et al., 2010). The key concept behind this algorithm is to use invariants to predict state of the gene expression pattern. We describe here how MiDReG and Boolean implication are used in B cell, bladder cancer, and colon cancer differentiation.

## B-Cell Differentiation

**Figure 2:**
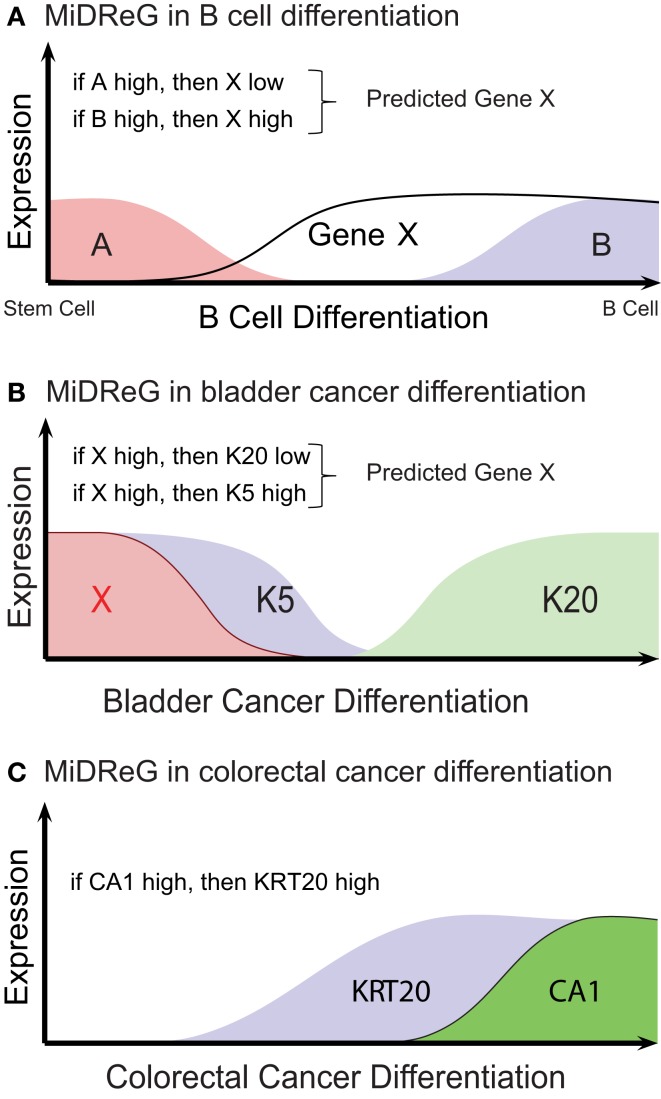
**Discovery of markers of differentiation using MiDReG algorithm**. Mining developmentally regulated genes (MiDReG) is an algorithm that uses Boolean implication to predict specific markers of differentiation in normal and cancer tissues. **(A)** MiDReG algorithm is used to predict markers of B-cell differentiation. **(B)** MiDReG algorithm is used to predict markers of bladder cancer differentiation. **(C)** MiDReG algorithm is used to predict markers of colorectal cancer differentiation.

B cells are special types of blood cell that are created from a blood stem cell by the process of differentiation. As the stem cell undergoes the process of differentiation, many genes changes their expression pattern. There are genes that are specific to the stem cell only and also there are genes that are specific to the differentiated B cell. MiDReG algorithm takes advantage of these gene pairs that have a significant Boolean implication “if A high, then B low,” and predict other genes that are expressed in the progenitors or precursors of B cells (Inlay et al., 2009; Sahoo et al., 2010). Let’s assume that gene A is expressed at the blood stem cells and it turns off as the stem cells differentiate to B cell. Similarly, let’s assume that gene B is off at the stem cell and it turns on as the stem cell differentiates to B cells (Figure 2A). Therefore, in this narrow view of differentiation gene A and gene B are mutually exclusively expressed. Let’s assume that there is a significant Boolean implication “if A high, then B low.” The significant Boolean implication represents a global invariant in all microarray datasets. In this case, if we want to identify a gene X that turns on after gene A turns off and before gene B turns on, we could simply use Boolean implication “if A high, X low,” and “if B high, X high” (Figure 2A). Since the Boolean implication is an invariant, we could hypothesize a state of differentiation where gene A is off, gene X is on, and gene B is off. In addition, this state of differentiation is between stem cell and the mature B cell. Therefore, gene X could potentially mark precursors of the mature B cell. We validated the gene expression patterns of the newly discovered genes using this approach by qPCR on the sorted B-cell progenitors from mouse blood and bone marrow. Review of the published literature of knockout mice revealed that many of our discovered genes were directly involved in B-cell differentiation. Out of 62 MiDReG genes, 41 genes were found to be knocked out in mice. Out of these 41 mice knockouts, 26 (63.4%) genes show defects in B-cell function and differentiation, 9 (22.0%) genes are associated with known B-cell function according to other experiments, and 6 (14.6%) genes could have a B-cell function based on their expression in the B cell and reported other hematopoietic functions. A detailed analysis on mouse lineages using MiDReG revealed a new earliest marker of B-cell differentiation Ly6D. This gene was investigated in detail by Inlay et al. (2009). Overall, our results on the B-cell differentiation suggested that MiDReG is a simple but extremely powerful approach to discover novel markers of progenitor cells.

## Bladder Cancer Differentiation

Differentiation within cancer is a very controversial topic (Reya et al., 2001). However, in bladder cancer it is established that there are two different cell types identified by Keratin 5 and Keratin 20 (Chan et al., 2009). Keratin 5 marks immature cell types that can differentiate to Keratin 20 positive cells (Chan et al., 2009). MiDReG algorithm was used to identify an upstream marker Keratin 14 (Volkmer et al., 2012). There is a significant Boolean implication relationship between Keratin 5 and Keratin 20 “if Keratin 5 high, then Keratin 20 low” that enabled the MiDReG algorithm to predict upstream markers. In this case, we are interested in a marker X that goes down early compared to Keratin 5. Thus, it is expressed at the most immature state of the cancer cell. The candidate markers were chosen based on Boolean implication “if X high, then Keratin 5 high” and “if X high, then Keratin 20 low” (Figure 2B). Keratin 14 was one of the markers that satisfied these two Boolean implication strongly. In addition, Keratin 14 was a single prognostic marker in both gene and protein expression datasets. The prognostic power of Keratin 14 was independent of currently established stage and grade. Therefore, a simple immunohistochemical analysis can identify high risk bladder cancer patients. Since, clinicians decide whether to perform cystectomy which is complete bladder removal based on stage and grade, it is possible to incorporate Keratin 14 based risk stratification into this important clinical decision endpoint. Clinicians are currently developing risk-adapted clinical strategies based on Keratin 14 for bladder cancer patients.

## Colon Cancer Differentiation

Many important markers in the differentiation of colon cancer cells follow Boolean implication (Dalerba et al., 2011). For example, there is a significant Boolean implication between Keratin 20 and CA1 “if CA1 high, then Keratin 20 high” (Figure 2C). This relationship is particularly strong with no exception. There are no tumors with CA1 high and KRT20 low. Even in a tumor when CA1 positive cells are present they have to go through a KRT20 positive precursor cell during differentiation. Accordingly, CA1 positive cells are a subset of Keratin 20 positive cells in both normal colon and colorectal cancer tissues. In addition, Keratin 20 negative patients have worse outcome compared to CA1 positive and Keratin 20 positive cancer patients. Other markers such as MS4A12, CD177, and SLC26A3 follow similar Boolean implication relationships.

## Strengths and Limitations

In this review we show that Boolean implication can be used to identify markers of differentiation in both normal and cancer tissues. The strength of Boolean implication is its ability to identify asymmetric gene expression relationships. In contrast, most other approaches focus on using symmetric gene expression relationship to build gene expression network. We have shown that some of the gene expression patterns in differentiation can be modeled using asymmetric Boolean implication. Therefore, it would be useful for predicting important genes involved in the process of differentiation. In addition, markers of differentiation are most likely robust prognostic biomarkers in cancer patients. Using these markers, clinicians may be able to develop better risk-adapted treatment decisions for cancer patients. The limitation of Boolean implication is that it requires large number of samples. Also, it might miss many other important genes that are involved in differentiation but do not have significant Boolean implication. Accordingly, Boolean implication is a very stringent criterion. Therefore, it pulls out many important genes and appears to be less noisy compared to traditional approaches.

An important distinction between Boolean implication analyses compared to other traditional network-based analyses is that most of these other analyses are focused on identifying gene regulatory networks or signal transduction pathways. Boolean implication has not been utilized to identify gene regulatory networks or signaling networks which contains simple feed-back and feed-forward structure. Instead, it was used to identify cell type or tissue specific gene expression patterns and they are interpreted in terms of development and differentiation. This is very different from Bayesian or mutual information based networks that primarily identify transcription factors and their targets (Segal et al., 2003; Basso et al., 2005; Margolin et al., 2006; Lee et al., 2009). Similarly, Boolean implication analyses are also different from traditional Boolean networks that are used to build a functional executable model or a circuit model (Glass and Kauffman, 1973; Shmulevich and Kauffman, 2004). There are also networks based on ODE models which describes mechanistic biochemical interactions (Ferrell et al., 2011). Both the Boolean and ODE based approaches described above models non-linear dynamical systems (Glass and Kauffman, 1973; Shmulevich and Kauffman, 2004; Ferrell et al., 2011). In contrast, Boolean implication analyses models static invariant relationships in a large biological dataset.

In summary, Boolean implication is an empirically observed relationship in the data, which may not hold for data gathered for different tissue types or under different conditions. Like correlation networks, Boolean implication networks do not capture causality. Boolean implication captures both symmetric as well as asymmetric relationships. It provides a powerful platform for discovery of novel markers of differentiation in both normal and cancer tissues.

## Conflict of Interest Statement

The author declares that the research was conducted in the absence of any commercial or financial relationships that could be construed as a potential conflict of interest.
